# Processing *α*-Chitin into Stable Composite Materials for Heavy Metal Adsorption

**DOI:** 10.3390/ijms26073149

**Published:** 2025-03-28

**Authors:** Anjana Aravind, Kristina Seliverstova, Kaitlin K. K. Kammerlander, Thomas Henle, Eike Brunner

**Affiliations:** 1 Bioanalytische Chemie, Fakultät für Chemie und Lebensmittelchemie, TU Dresden, 01062 Dresden, Germany; anjana.aravind@tu-dresden.de (A.A.);; 2 Lebensmittelchemie, Fakultät für Chemie und Lebensmittelchemie, TU Dresden, 01062 Dresden, Germany; kristina.seliverstova@tu-dresden.de (K.S.); thomas.henle@tu-dresden.de (T.H.)

**Keywords:** *α*-chitin, N-acetyl-D-glucosamine, ionic liquids, composite, characterisation, europium adsorption

## Abstract

Water contamination by heavy metals, including radionuclides, is a major threat to human health and the environment. New methods for their removal are therefore needed. Adsorption is currently a common method for wastewater treatment. It depends on the physical and chemical interactions between heavy metal ions and adsorbents. The main characteristics of suitable adsorption methods are (i) a high adsorption efficiency and ability to remove different types of ions, (ii) a high retention time and cycle stability of adsorbents, and (iii) availability. Chitin is a commercially available biopolymer from marine waste that has several favourable properties: availability, low cost, high biocompatibility, biodegradability, and effective adsorption properties for metal ions. However, the processing of chitin into stable structures, such as chitin-based composites, is difficult due to its high chemical stability and extremely low solubility in most solvents. The central working hypothesis of the present work is that powdered *α*-chitin can be dissolved in the ionic liquid 1-butyl-3-methylimidazolium acetate and cross-linked with its monomer, N-acetyl-D-glucosamine, in a Maillard-like or caramelisation reaction to produce chitin-based composites. It is further hypothesised that such composites can be used as biosorbents for heavy metal ions. Eu(III) is chosen here as a non-radioactive representative and analogue for other f-elements.

## 1. Introduction

Mining activities as well as nuclear energy production lead to the risk of the release of heavy metals, including radionuclides, into the environment as well as food chains. Many heavy elements are toxic. Long-living radionuclides (actinides) are of further concern due to their radioactivity. Given their high toxicity and carcinogenicity, heavy elements and especially actinides pose severe risks to human health as well as ecosystems. There are several methods for their removal from aqueous solutions, such as solvent extraction, ion exchange, coagulation, electrodeposition, and reverse osmosis. Currently, adsorption attracts increasing attention due to its high efficiency, simplicity, and low cost [1,2,3,4,5,6,7]. Adsorption using functional materials has been demonstrated to be an efficient technique for removing heavy metals/radionuclides from water [8,9,10,11,12]. There is already a market for low-cost materials with a high adsorption capacity under intense environmental conditions to remove radionuclides.

Chitin (C_8_H_13_O_5_N)_n_ is the second most abundant biopolymer after cellulose. In the chemical sense, it is a polysaccharide consisting of *β*-1,4-linked N-acetyl-D-glucosamine (GlcNAc) units [13,14]. It is a primary component of the cell walls in fungi and of the exoskeletons in various invertebrates such as crustaceans and insects [13], and even in some marine sponges [15]. Chitin is also produced by several other living organisms in the lower plant and animal kingdoms, serving many functions like reinforcement/mechanical strength [16]. Chitin is well-suited for various applications due to its excellent biocompatibility, biodegradability, hydrophilicity, low toxicity, and availability from the aforementioned renewable resources. Depending on its source, chitin occurs as three allomorphs: the *α*, *β*, and *γ* forms, which can be differentiated, e.g., by attenuated total reflectance (ATR), Fourier transform infrared (FTIR), and solid-state nuclear magnetic resonance (NMR) spectroscopy, as well as X-ray diffraction [17]. *α*-chitin is the stable and most abundant form. It occurs in fungal and yeast cell walls, krill, lobster, crab tendons, shrimp shells, insect cuticles, and even in the skeletons of some marine sponge species [7]. Hence, *α*-chitin is a low-cost and easily available biopolymer. Its use as an eco-friendly material is thus desirable from the point of view of sustainability.

Chitin is also well-known for its favourable adsorption properties, especially with respect to various heavy metal ions from solutions, including uranium, cadmium, iron, nickel, copper, lead, and zinc [18,19,20,21,22,23,24]. This is due to suitable functionalities, especially carbonyl and hydroxyl groups. For example, Lin et al. [20] demonstrated efficient uranyl-carbonate removal from aqueous solutions by film-like chitin/polyethyleneimine (CH-PEI) biosorbents. Duan et al. [19] exploited a synergistic effect of chitin in combination with lignin, thus enabling more efficient adsorption of iron and copper. Boulaiche et al. [22] evaluated the effective biosorption of heavy metals such as copper, zinc, lead, and cadmium by chitin. However, the crystalline and stable *α*-chitin is unable to swell, and most of these functional groups are thus inaccessible for the metal ions in solution. Using a special, sponge-like form of *α*-chitin extracted from a marine sponge with a comparably high external surface area, very high adsorption capacities for uranyl ions from aqueous solutions could be obtained [18], which emphasises the need to enhance the external surface area. However, the primary sources of chitin are crab and shrimp shells. This material is commercially available as a powder consisting of *α*-chitin particles. The transformation of this commercially available material into stable chitin-based composites, sponge-like structures, or other composites would thus be highly desirable. However, chitin is hardly soluble by organic solvents or water due to its very stable, strongly hydrogen-bonded structure [17,25]. Dissolution of chitin is only feasible in very few solvents [17,26,27,28], which are, however, toxic, degradable, corrosive, scarce, or mutagenic. Therefore, most current uses of this natural resource are associated with chitosan, a soluble, partially or completely deacetylated derivative of chitin.

Recently, ionic liquids (ILs) have been documented to dissolve biopolymers and are considered as “green” solvents capable of substituting the volatile organic compounds (VOCs) generally used in different processing and synthesis industries. The pioneering work on the utilisation of ILs as solvents for polysaccharides was carried out by the group of Roger [25,29,30]. It was found that ILs could be employed as non-derivatising solvents for native cellulose. One of the ILs used in this work, 1-butyl-3-methylimidazolium chloride ([BMIM][Cl]), showed the best solvating capability. Up to 25 wt.% cellulose could be dissolved under microwave heating. That means, ILs obviously provide efficient access to this natural biopolymer and subsequently enable an entire product platform based on this renewable source, a significant step towards sustainability. Wu et al. [31] developed an approach to dissolve *α*-chitin in 1-butyl-3-methylimidazolium acetate [BMIM][OAc]. This IL was used for the dissolution processing of the biopolymer into a microsphere architecture.

Based on this preliminary work, the central working hypothesis of the present work is that powdered α-chitin can be dissolved in the ionic liquid 1-butyl-3-methylimidazolium acetate and can be cross-linked using its monomer, N-acetyl-D-glucosamine, in a Maillard-like or caramelisation reaction to produce chitin-based composites. It is also hypothesised that such composites can be used as mechanically stable adsorbents for heavy metal ions. Europium (Eu) is typically utilised as a non-radioactive analogue for other trivalent lanthanides and actinides because of its similar physicochemical properties [32]. The present study investigated the biosorption of trivalent Eu(III) on the processed chitin-based composites created from the commercially available biopolymer *α*-chitin. Their morphology, structure, and surface properties were analysed using FTIR, solid-state NMR spectroscopy, and SEM analysis methods. The biosorption of Eu(III) was quantified using inductively coupled plasma optical emission spectroscopy (ICP-OES).

## 2. Results and Discussion

### 2.1. Characterisation of Composites

**Figure 1:**
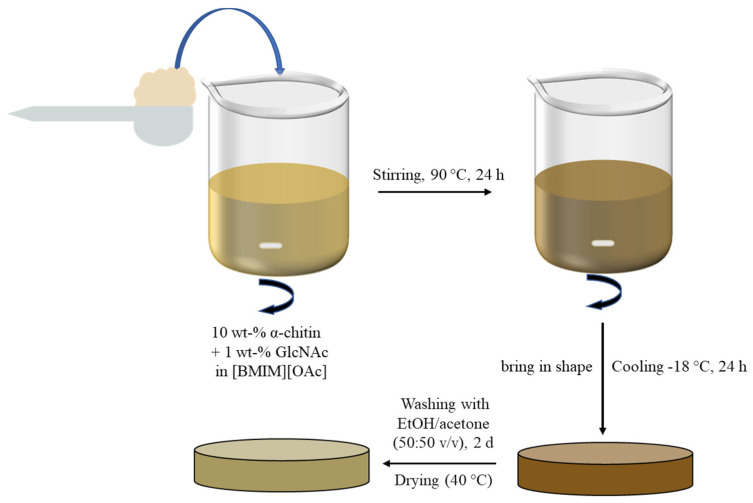
Experimental scheme of the synthesis procedure of chitin-based composites.

**Figure 2:**
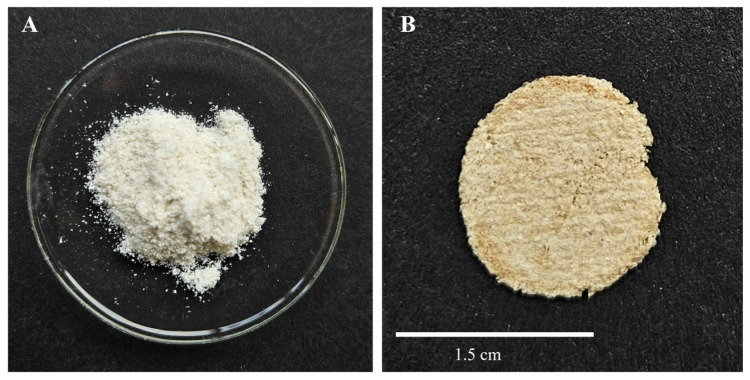
(**A**) Initial material (commercial α-chitin powder) and (**B**) prepared chitin-based composite after washing in EtOH/acetone and oven-drying.

The first step after the preparation and drying of the chitin-based composites (cf. Figure 1) was the visual inspection of the material (see Figure 2).

Figure 2 shows the commercial *α*-chitin and the synthesised chitin-based composite. It can already be seen visually that the oven-dried, chitin-based composite has an amber colour. This is characteristic for the reaction of sugars in non-enzymatic browning reactions such as caramelisation or the Maillard reaction [33,34]. This principle was used here for the preparation of the composite, which is a novel contribution of the work presented.

**Figure 3:**
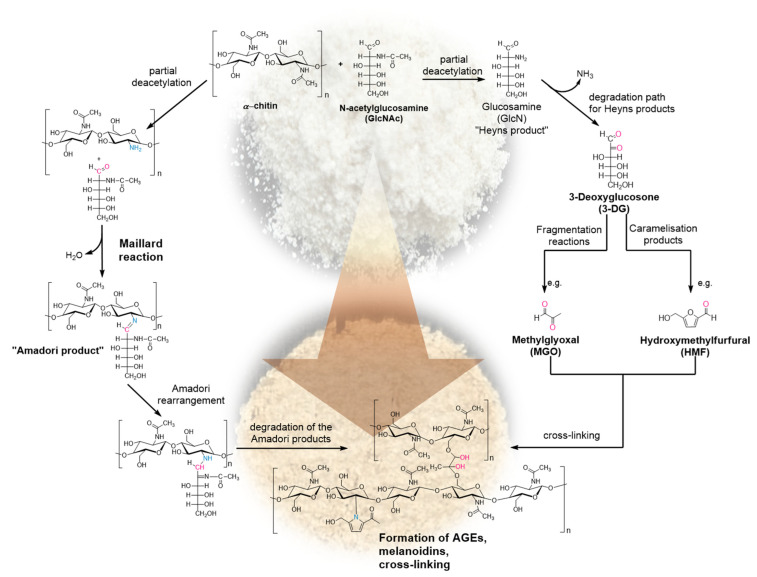
Proposed mechanism of interaction and cross-linking between *α*-chitin and GlcNAc via Maillard self-reaction and caramelisation processes.

In short, the reaction described by Louis-Camille Maillard in 1912 [35] is an amino-carbonyl reaction which is particularly important in processed food, such as baking bread or roasting coffee. Based on the above-mentioned examples, reducing sugars and amino components such as amino acids or proteins react with each other to produce a variety of odour- and taste-active products, as well as colouring components such as melanoidins and other “advanced glycation end products” (AGEs) [34]. The precursors of these end products include Amadori and Heyns products. Later dicarbonyls, e.g., methylglyoxal, are formed at different stages of the Maillard reaction. However, they can also be formed during the classical caramelisation of sugars [34]. The particular focus on such compounds is due to their potential as possible cross-linking agents. The idea is that the addition of the monomer GlcNAc can promote cross-linking between the individual polymer chains of chitin. This should lead to an increased stability but also flexibility of the composites. It is assumed that the reaction could lead to the formation of dicarbonyls, as well as to a reaction of the carbonyl group of the monomer with the partially diacetylated residues in the chitin. The latter would be described as a Maillard self-reaction [36,37]. The hypotheses for the formation of such cross-links are summarised in Figure 3.

**Figure 4:**
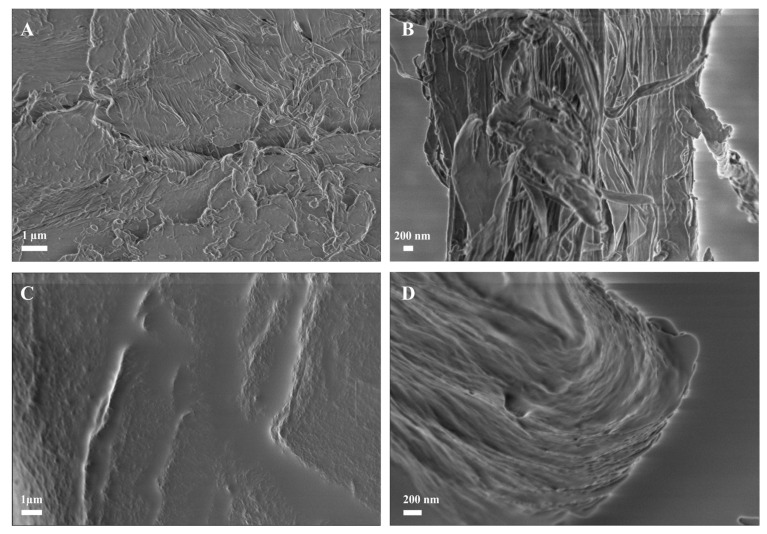
Morphological characterisation of the initial material (α-chitin) and produced chitin-based composites by SEM: (**A**) surface and (**B**) interior structure of dried α-chitin; (**C**) surface structure and (**D**) interior structure of formed and oven-dried, chitin-based composites.

Scanning electron microscopy was used for the morphological characterisation of the chitin-based composites. Figure 4 shows the images of commercial *α*-chitin compared to the prepared material. The structural difference is evident. While the native chitin powder has an uneven surface (A), the prepared composite appears to have a smooth and uniform surface with no visible particles (C). This indicates that chitin has been successfully dissolved and restructured during the manufacturing process. The lateral image of the fragments also shows a difference. The *α*-chitin has an unstructured, fibrous structure (B), whereas the chitin-based composite appears to have an ordered, layered structure (D). Similar observations were made by Wu et al. [31] for their composites obtained without the GlcNAc cross-linking agent.

**Figure 5:**
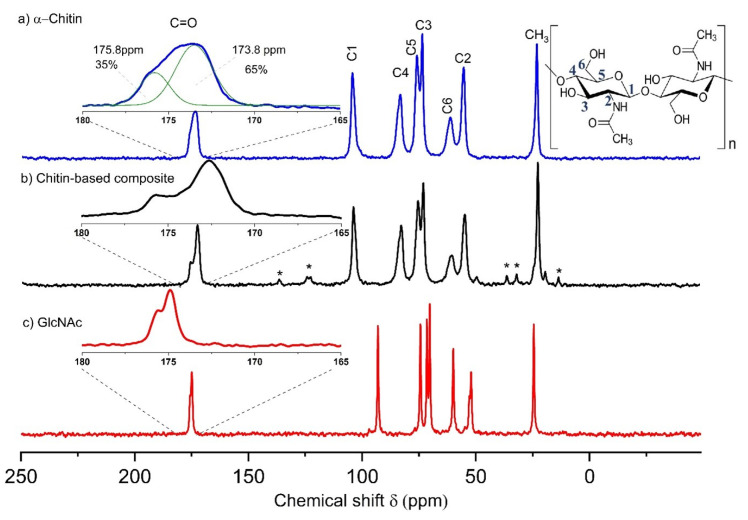
^13^C CP MAS NMR spectra of (**a**) α-chitin, (**b**) chitin-based composite, and (**c**) GlcNAc. CP contact time: 4 ms. The minor signals indicated by an asterisk in the composite spectrum are due to residual spurious amounts of the ionic liquid used for the processing. The insert in (**a**) shows the decomposition of the C=O signal into the two components discussed in text (green lines).

**Figure 6:**
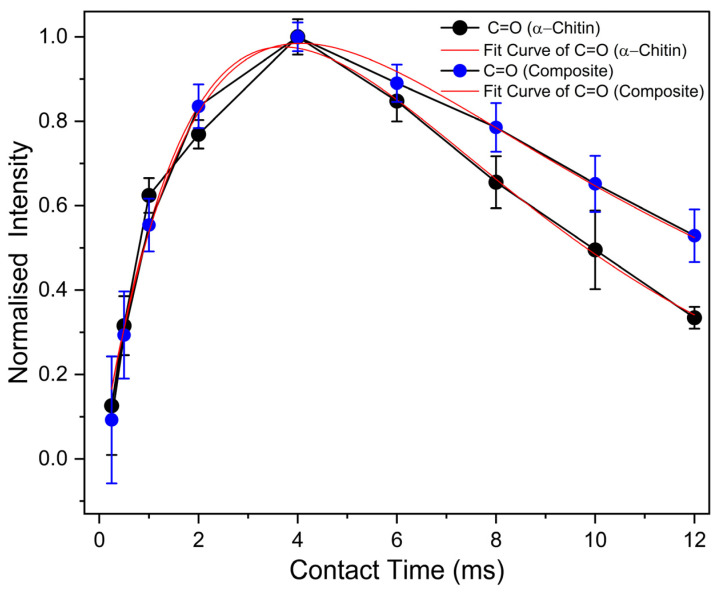
CP build-up curves for the C=O signal in *α*-chitin and the chitin-based composite measured at variable contact times of 0.25–12 ms. Furthermore, the fitted curves are shown, which allow the determination of *T*_CP_ and *T*_1*ρ*_.

In order to characterise the materials at the molecular level, ^13^C cross-polarisation (CP) MAS NMR experiments were performed. Figure 5 shows the ^13^C CP MAS NMR spectra of *α*-chitin, the chitin-based composite, and monomeric GlcNAc. The spectra of the samples show well-resolved signals from each expected carbon position (see structure inserted in Figure 5). The signal assignment for *α*-chitin following the literature [38] is given in Table S1 (ESI). The spectra measured for the *α*-chitin reference samples agree very well with previously published data [38,39,40,41]. Kameda et al. [39] demonstrated that the carbonyl ^13^C NMR signal at room temperature consists of two lines due to different hydrogen bonds in *α*-chitin. At elevated temperature, these two signals merge into a single, symmetric peak, indicating an exchange between the carbonyl carbons involved in different hydrogen-bonding environments. The spectrum of *α*-chitin measured here at room temperature also shows the presence of these two components (Figure 5a). The C=O signal of N-acetyl-D-glucosamine (Figure 5c) exhibits a small splitting due to the residual dipolar coupling with the neighbouring quadrupolar ^14^N nucleus (*S* = 1). The magnetic dipole–dipole interaction between a spin-1/2 nucleus and a nucleus carrying an electric quadrupole moment (i.e., a spin with S > 1/2) can result in a characteristic residual dipolar broadening and splitting of the MAS NMR signals of the spin-1/2 nucleus [42,43]. The main difference between *α*-chitin and the chitin-based composites occurs in the region C=O carbon (see inserts in Figure 5a,b). The signal from chitin shifts to lower chemical shift values and becomes narrower. Note that the less intense peak at the higher chemical shift is likely due to the cross-linking of the monomer unit. This observation confirms the observations described above in the context of Figure 3: the cross-linking reaction takes place via caramelisation or the Maillard reaction [33,36,37], whereby the N-acetyl-D-glucosamine moiety seems to be the interaction site. Further support for this idea comes from the cross-polarisation (CP) kinetics of ^1^H-^13^C CP MAS NMR experiments [44,45]. Depending on the dipolar coupling strength, the ^13^C NMR signals build up and decay at different rates. Mobility also affects the CP efficiency. The intensities of the ^13^C CP MAS NMR signals were measured at different contact times ranging from 0.25 ms to 10 ms. The resonance peak intensity follows the function described in the literature (see, e.g., [46]). The variation in the contact time allows the determination of both the cross-polarisation build-up time (*T*_CP_) and proton spin-lattice relaxation time in the rotating frame (*T*_1ρ_). Figure 6 shows the intensity (peak area) of the ^13^C CP MAS NMR signal of C=O as a function of the contact time for *α*-chitin and the composite. All the cross-polarisation build-up times *T*_CP_ and proton spin-lattice relaxation times *T*_1*ρ*_ of *α*-chitin and the composite are given in Figure S3 (ESI). Significant differences between *α*-chitin and the composite occur selectively for the C=O signal, especially for *T*_1*ρ*_. This observation further indicates that the cross-linking with the monomer mainly occurs via the GlcNAc moiety of chitin.

**Figure 7:**
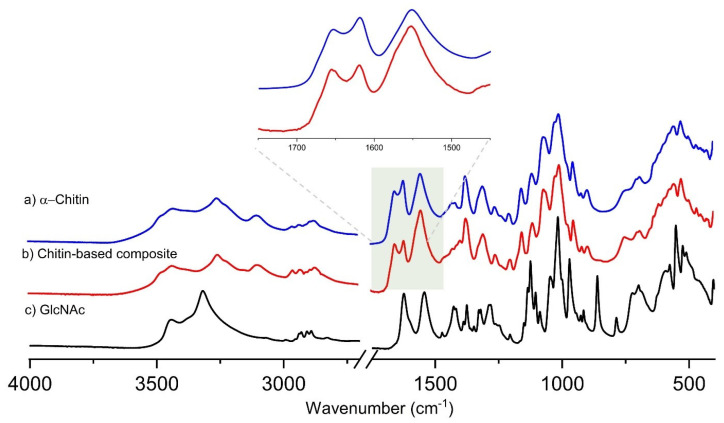
ATR-FTIR spectra of α-chitin (**a**), chitin-based composite (**b**), and GlcNAc monomer (**c**).

The infrared spectra are displayed in Figure 7. The bands are assigned based on the literature [13,15,47] (see Table S2 (ESI)). IR spectroscopy can distinguish the different chitin crystal structures (*α* or *β*) due to the influence of the different hydrogen bond patterns upon the spectra [13]. Two amide bands at 1653 and 1619 cm^−1^ [48] occur due to two different hydrogen bond states. The component at 1653 cm^−1^ is assigned to C=O groups hydrogen bonded only to NH groups, while the component at 1619 cm^−1^ should be ascribed to the similar group with another hydrogen bond to the side chain CH_2_OH [49]. The existence of these inter-chain hydrogen bonds is responsible for the high chemical stability of the α-chitin structure [13]. In the chitin-based composites, these two bands are narrower and have slightly different relative intensities in line with the conclusions drawn above from the ^13^C CP MAS NMR spectra.

### 2.2. Characterisation of the Adsorbent After Eu Adsorption

**Figure 8:**
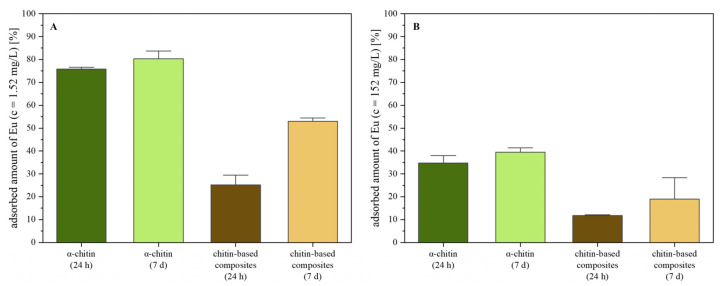
Characterisation of the europium adsorption on chitin and chitin-based composites by ICP-OES: (**A**) adsorbed europium amount after 24 h and 7 d [c(EuCl_3_∙6H_2_O) = 10^−5^ M] and (**B**) adsorbed europium amount after 24 h and 7 d [c(EuCl_3_∙6H_2_O) = 10^−3^ M].

The ICP-OES studies reveal (Figure 8) that the pure *α*-chitin adsorbed more europium than the composite for both initial Eu(III) concentrations in solution. With increasing time, the adsorption capacity increases for both materials.

Pure chitin is very efficient in adsorbing the small amount of Eu(III) dissolved in the solution with a 10^−5^ M concentration, which corresponds to only 1.52 mg/L. The composites are less efficient. Their decreased adsorption capacity is probably due to the partial blocking of adsorption sites by cross-linking with the monomers via the probable interaction sites, which are the C=O (see above). For the highly concentrated solution (10^−3^ M Eu(III) corresponding to 152 mg/L), the trend is similar. The adsorption capacity, i.e., the amount of Eu(III) adsorbed per gram of adsorbent material, is calculated using Equation (1). The adsorption capacity of *α*-chitin after 24 h and seven days and the 10^−3^ M solution was 33 mg/g (milligram europium per gram material) and 38 mg/g. For the composite, 11 mg/g and 18 mg/g europium loading are obtained after 24 h and seven days. The comparison shows that native *α*-chitin has a higher adsorption capacity for Eu(III) than the composites at both adsorption times.

To ensure the structural integrity of the adsorbents after europium sorption, we measured ^13^C CP MAS NMR and ATR-IR spectra (see ESI, Figures S1 and S2) and compared them with the spectra of the unloaded samples discussed above (Figure 5 and Figure 7). The adsorption of europium did not result in significant changes in the spectra apart from decent line broadening in the NMR spectra, which is likely due to the paramagnetic europium ions. This indicates that the europium ion is not covalently bound to chitin, as the formation of bonds should be visible in the NMR spectra in the form of measurable chemical shifts or signal splitting. The absence of observable changes in the measured spectra strongly implies that europium caused no chemical modification or destruction of the chitin. A probable interaction mechanism that may explain all these observations is the coordination of europium via the C=O groups. This may also involve the formation of hydrogen bonds between the hydroxyl groups of the different europium complexes and the polymer. Such weak interactions tend to favour the reversible reaction and the easy desorption of the metal. That means, the overall configuration of the polymer chain is maintained during the process.

## 3. Materials and Methods

### 3.1. Chemicals

The chitin materials used in this study include *α*-chitin from crab shells purchased from Carl Roth (Karlsruhe, Germany) of 400,000 g/mol molecular mass, respectively. EuCl_3_·6H_2_O (99.99%) and N-acetyl-D-glucosamine (≥95%, MW: 221.2 g/mol) were purchased from Sigma Aldrich (St. Louis, MO, USA). The chitin and N-acetyl glucosamine were used directly without further purification. 1-Butyl-3-methyl-imidazolium acetate [BMIM][OAc] was purchased from Iolitech (Heilbronn, Germany) at ≥98% purity. Ethanol (EtOH) and acetone of high-performance liquid chromatography (HPLC) grade were obtained from Fisher Chemicals (Schwerte, Germany).

### 3.2. Sample Preparation

The preparation of the chitin-based composites follows a modified procedure inspired by the previous work of Wu et al. [31]. In contrast to previous studies, the monomer N-acetyl-D-glucosamine was added to α-chitin for the first time here as a cross-linker. A 10 wt.-% solution of α-chitin and 1 wt.-% of GlcNAc in IL was prepared by dissolving 0.3 g of chitin and 0.03 g of GlcNAc in 3 mL of [BMIM][OAc]. Chitin, GlcNAc, and IL were mixed and heated in an oil bath at 90 °C (±5 °C) for 24 h until full dissolution of the chitin. During heating and stirring, the solution became an amber colour. After 24 h, the hot solution was poured over a round-shaped Teflon form with an inner diameter of 17 mm and 2.3 mm depth. After shaping and cooling to room temperature, the composite was cooled overnight at −20 °C. Afterwards, the composites were placed in a solution of ethanol and acetone to remove the IL. After washing, the finished chitin-based composites were dried at 40 °C in a drying oven for 2 days. These treatment steps are schematically summarised in Figure 1.

### 3.3. Batch Eu(III) Sorption Experiments

A total of 15.5 mg (±0.3 mg) of the chitin and chitin-based composite was suspended in 10 mL of solution with either 10^−3^ mol/L or 10^−5^ mol/L EuCl_3_∙6H_2_O in ultrapure water. Pure α-chitin was loaded with europium for comparison. The suspensions were shaken in total for 7 days; however, an aliquot was taken after 24 h. Since the central goal of our present work was to develop a synthesis procedure for stable composites that maintain the favourable biosorption properties of pure chitin at least partly, it was decided to evaluate the short-term behaviour after 24 h and the long-term behaviour after 7 days. Afterward the samples were centrifuged at 5000 rpm for 10 min, the supernatant was separated from the pellet and used for ICP-OES. The pellet of the sample was dried under air atmosphere, and the Eu(III) uptake (*q*) was calculated as follows [20]:

(1) q=C0−Ci ·Vm

where *C*_0_ and *C_i_* (mg/L) are the concentrations of Eu(III) before and after adsorption, respectively; *V* is the volume of Eu(III) solution; and *m* is the weight of the adsorbent (α-chitin or chitin-based composite).

### 3.4. Scanning Electron Microscopy (SEM)

Scanning electron microscopy (SEM) was used to examine the sample morphologies. The surface morphology of chitin and chitin-based composites samples was recorded at room temperature using a scanning electron microscope, Oxford XMaxN 150—150 mm^2^, (Oxford Instruments, Abingdon, UK) with an acceleration voltage of 50 kV, accessible through the Dresden Center for Nanoanalysis (DCN) at TU Dresden.

### 3.5. Fourier Transform Infrared (FTIR) Spectroscopy

The FTIR measurements were performed on a Thermo Scientific Nicolet iS5 spectrometer (Waltham, MA, USA) equipped with a broad-band mercury-cadmium-telluride detector. The spectra were acquired in the attenuated total reflection (ATR) mode using single-reflection monolithic durable diamond ATR Specac’s Golden Gate accessory (Orpington, UK). All spectra were acquired from a spot 2 mm in diameter on samples pressed against the diamond crystal. Spectra (500 scans at 2 cm^−1^ resolution) were collected in the 4000–400 cm^−1^ range.

### 3.6. Nuclear Magnetic Resonance (NMR)

Solid-state ^13^C nuclear magnetic resonance (NMR) spectra were acquired using a Bruker Ascend 300 MHz NMR spectrometer at 75.47 MHz for ^13^C using a commercial double-resonance 4 mm magic-angle spinning (MAS) NMR probe (Bruker Biospin, Ettlingen, Gemany). Approximately 15 mg of sample was packed into the rotors and spun at 15 kHz. Ramped cross-polarisation (CP) was used for all the samples spun at the magic angle of 15 kHz. A CP contact time of 4 ms and a recycle delay of 3 s were used. Free induction decays (FIDs) were accumulated (in total 26,000 scans) with a total acquisition time of 22 h. The spectral width was 30 kHz. Furthermore, CP build-up curves were acquired with a contact time variation between 0.25 and 10 ms. Spectra were referenced relative to tetramethylsilane (TMS) using adamantane with its well-known signals at 29.5 ppm and 18.5 ppm as the secondary standard.

### 3.7. Inductively Coupled Plasma Optical Emission Spectroscopy (ICP-OES)

To analyse the sorption properties of the chitin-based composites and the pure chitin, the supernatant from the batch adsorption tests was measured and compared with the initial concentration of the Eu(III) solution. Therefore, samples were diluted in HNO_3_ (5%), respectively. The resulting liquids were analysed with an Optima 7000DV spectrometer (Perkin Elmer, Waltham, MA, USA) utilising the following parameters: high frequency power 1300 W, liquid flow 1.6 L/min, plasma gas flow 15 L/min, auxiliary gas flow 0.2 L/min, and nebuliser gas flow 0.65 L/min. The spectral line was 412.970 nm for the radial detection of europium.

## 4. Conclusions

The working hypothesis of the present study was confirmed: we have successfully established a novel method for processing commercially available powdered α-chitin into stable composites based on dissolution in the IL 1-butyl-3-methylimidazolium acetate and cross-linking with the added monomer N-acetyl-D-glucosamine at relatively low temperatures. After cooling these solutions to room temperature, gels remain, and the chitin-based composites are then obtained by ethanol/acetone coagulation. Cross-linking with GlcNAc in a Maillard-like or caramelisation reaction gives mechanically stable composites for applications such as filter materials. FTIR studies the structural integrity of the chitin after these processing steps. ^13^C solid-state NMR experiments showed changes in the NMR parameters of the C=O signal induced by cross-linking with the GlcNAc monomer. This implies that the N-acetyl moiety of chitin is influenced by the cross-linking. The suitability of these composites for heavy metal biosorption was demonstrated using europium as an analogue for f-elements, confirming a second working hypothesis of our present work. Europium adsorption did not lead to any changes in the spectra indicative of chemical modifications of the chitin or the composites. The ICP-OES data showed that the original α-chitin biosorbed more Eu(III) than the chitin-based composites at different concentrations. It appears that the cross-linking monomer partially blocks the adsorption sites, which are probably the C=O groups.

In summary, commercially available chitin is successfully processed into mechanically and chemically stable composites with promising properties. Future work should focus on further improving the biosorption properties, e.g., by using other cross-linking agents that do not block the C=O groups or—ideally—even providing other functional groups that provide additional interaction sites for metal ions to improve the adsorption properties.

## Data Availability

The original contributions presented in this study are included in the article and Supplementary Materials. Further inquiries can be directed to the corresponding author.

## Supplementary Materials

The following supporting information can be downloaded at: https://www.mdpi.com/article/10.3390/ijms26073149/s1.
