# Supplementary material for: Processing α-Chitin into Stable Composite Materials for Heavy Metal Adsorption
Source: Int J Mol Sci. 2025 Mar 28;26(7):3149. doi: 10.3390/ijms26073149 (PMC11988694; doi:10.3390/ijms26073149)
Supplement: Supplementary file 1 [file ijms-26-03149-s001.zip › ijms-3548729-supplementary.pdf]

Electronic Supporting Information

for

Processing *α*-Chitin into Stable Composite Materials for Heavy Metal Adsorption

Anjana Aravind [1†], Kristina Seliverstova [2†], Kaitlin K. K. Kammerlander[1], Thomas Henle[2], Eike Brunner[1]

[1] TU Dresden, Fakultät für Chemie und Lebensmittelchemie, Bioanalytische Chemie, 01062 Dresden, Germany; eike.brunner@tu-dresden.de; anjana.aravind@tu-dresden.de

[2] TU Dresden, Fakultät für Chemie und Lebensmittelchemie, Lebensmittelchemie, 01062 Dresden, Germany; thomas.henle@tu-dresden.de; kristina.seliverstova@tu-dresden.de

[†] These authors contributed equally to the present work.

***** Correspondence: eike.brunner@tu-dresden.de

1. ^{13}C cross polarization/magic angle spinning nuclear magnetic resonance (CP MAS NMR) spectra of the adsorbent before and after Eu(III) biosorption

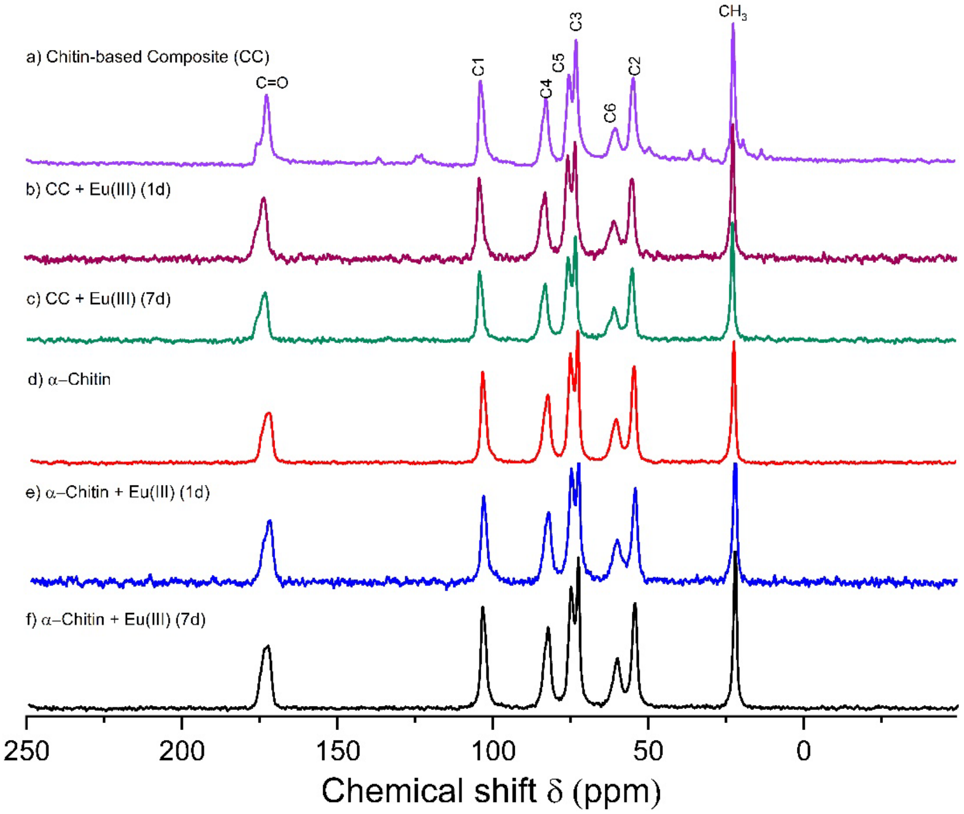

Figure S1. ^{13}C CP MAS NMR spectra of (a) chitin-based composite, (b) after Eu(III) adsorption for 1d, (C) after 7d Eu(III) adsorption, (d) pure α-chitin, (e) after 1d Eu(III) adsorption, (f) after 7d Eu(III) adsorption.

The signals of *α*-chitin and the chitin-based composites (CC) were assigned according to literature [1] and are summarized in Table S1:

Sample	C=O		C-1		C-4		C-5		C-3		C-6		C-2		CH_3	
	δ (ppm)	$\Delta\nu_{1/2}$ (Hz)	δ (ppm)	$\Delta\nu_{1/2}$ (Hz)	δ (ppm)	$\Delta\nu_{1/2}$ (Hz)	δ (ppm)	$\Delta\nu_{1/2}$ (Hz)	δ (ppm)	$\Delta\nu_{1/2}$ (Hz)	δ (ppm)	$\Delta\nu_{1/2}$ (Hz)	δ (ppm)	$\Delta\nu_{1/2}$ (Hz)	δ (ppm)	$\Delta\nu_{1/2}$ (Hz)
α-Chitin	174	211.9	104.1	123.1	83.5	175.2	75.7	151.3	73.7	109.8	61.2	228.9	55.4	124.3	23	83.6
α-C(1d)	173.8	196.0	104.2	135.5	83.5	179.5	75.7	173.2	73.7	130.0	61.0	260.8	55.3	140.8	22.9	92.2
α-C(7d)	174.0	214.3	104.2	126.5	83.4	167.8	75.8	157.7	73.8	112.1	61.0	221.1	55.4	133.2	23.0	79.7
CC	173.1	175.2	103.9	122.3	83.4	190	75.4	169.7	73.4	123.4	61.0	252.4	55.3	133.6	22.9	104.9
CC(1d)	173.6	194.2	104.1	131.2	83.4	180.5	73.8	159.7	73.7	129.6	61.0	285.8	55.3	141.8	22.9	89.9
CC(7d)	173.8	97.4	104.2	122.7	83.5	166.1	85.8	109.9	73.8	156.4	61.1	242.3	55.4	129.9	23.0	79.9

Table S1. Chemical shifts and line widths of the ^{13}C NMR signal observed for the α-chitin and the chitin-based composite before and after Eu(III) biosorption

2. ATR-FTIR spectra of absorbents before and after Eu(III) biosorption with band assignment

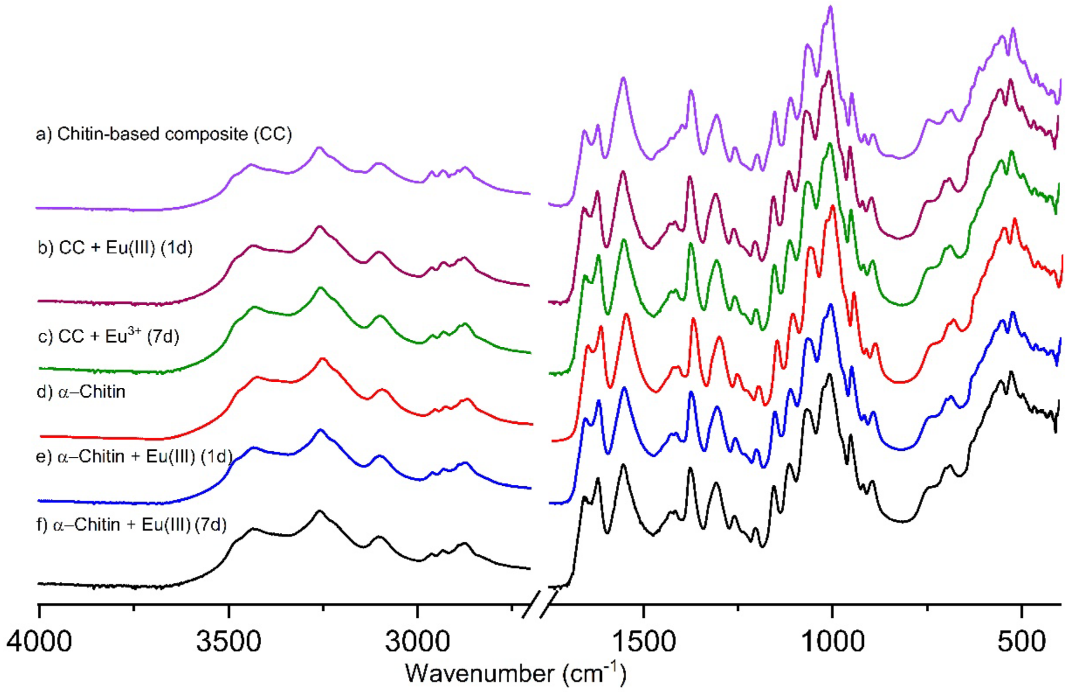

Figure S2. ATR-FTIR spectra of spectra of (a) chitin- based composite, (b) after Eu(III) adsorption for 1d, (C) after 7d Eu(III) adsorption, (d) pure α-chitin, (e) after 1d Eu(III) adsorption, (f) after 7d Eu(III) adsorption.

The IR bands are assigned based according to the literature [2-5] and are summarized in Table S2:

Assignment	α-chitin λ(cm^{-1})	Film λ(cm^{-1})	α-chitin(1d) λ(cm^{-1})	α-chitin(7d) λ(cm^{-1})	film(1d) λ(cm^{-1})	film(7d) λ(cm^{-1})
ν_{OH}	3432	3433	3431	3435	3433	3431
ν^{as}_{NH}	3255	3257	3256	3255	3256	3255
ν^{s}_{NH}	3099	3101	3095	3096	3098	3098
ν Aliphatic CH_x, s	2961-2876	2960-2874	2960-2872	2959-2872	2960-2873	2960-2873
$\nu_{C=O}$ (Amide I)	1653,1619	1653,1617	1656, 1616	1656, 1619	1653, 1618	1653, 1618
$\nu_{C\text{-}N}$(C-N-H) +δ_{NH} (Amide II)	1552	1552	1552	1552	1552	1552
δ_{CH2}	1427	1427	1427	1426	1427	1526
ν_{CH}+δ_{NH} (Amide III)	1306	1306	1307	1306	1307	1307
δNH	1260	1258	1257	1259	1258	1259
$\nu_{C\text{-}O\text{-}C}$, $\nu_{C\text{-}O}$	1154-1007	1154-1007	1154-1007	1154-1007	1157-1007	1154-1007
Υ_{CH3} d	895	895	894	894	893	895

Table S2. Wavenumbers and assignment of the bands observed in the FTIR spectra of the α-chitin and the chitin- based composite before and after Eu(III) biosorption.

3. Analysis of cross polarization (CP) build-up curves

The CP build fit was taken from equation in literature [6] considering that the transfer of nuclear spin polarization between two spin systems follows first-order kinetics.

$$S(t) \sim \left(\frac{1}{1-\left(T_{CP}/T_{1\rho}\right)}\right)\left[1 - exp\left(-\frac{\left(1-T_{CP}/T_{1\rho}\right)t}{T_{CP}}\right)\right] exp\left(-\frac{t}{T_{1\rho}}\right)$$

Here,

$S(t)$: Nuclear spin polarization at a given time t

T_{CP}: CP build-up time constant

$T_{1\rho}$: Longitudinal relaxation time in the rotating frame

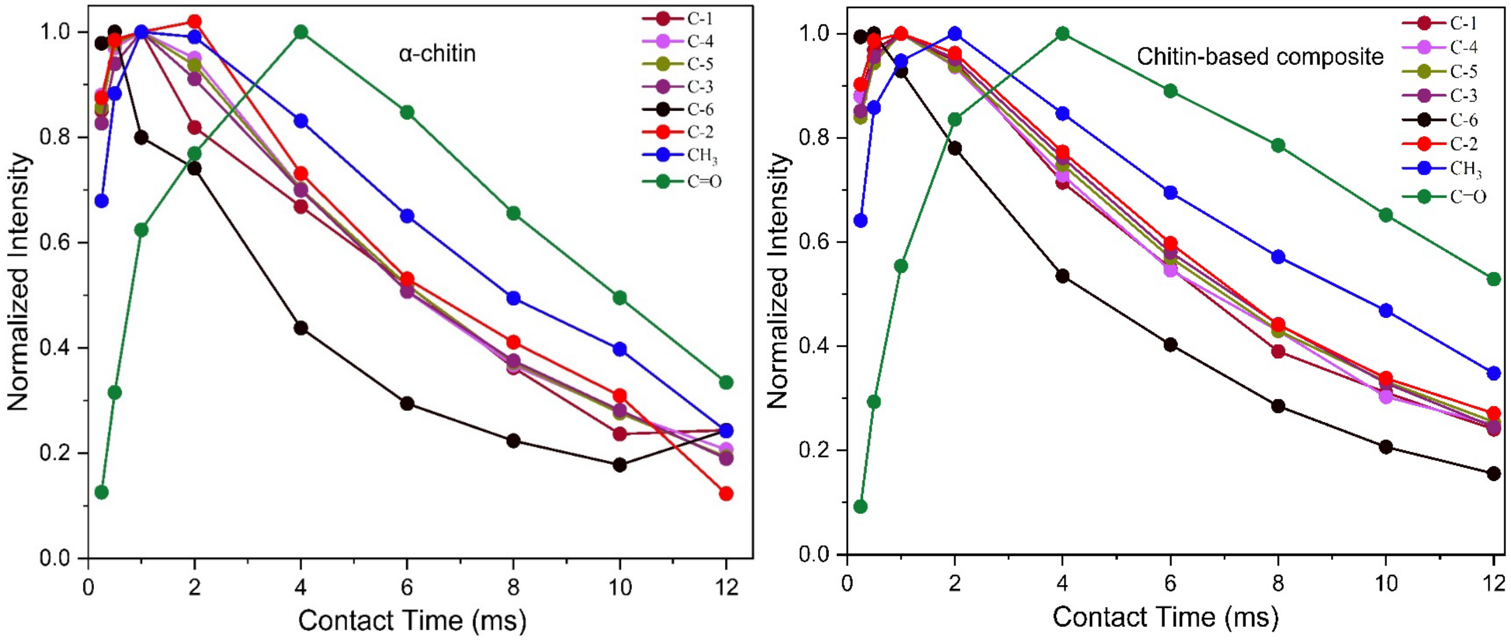

Figure S3: ^{13}C CP build-up curves for all the signals in α-chitin and chitin-based composite (CC) measured at variable contact times 0.25 – 12 ms.

References

1. Belton, P.S.; Tanner, S.F.; Cartier, N.; Chanzy, H. High-Resolution Solid-State Carbon-13 Nuclear Magnetic Resonance Spectroscopy of Tunicin, an Animal Cellulose. *Macromolecules* **1989**, *22*, 1615–1617. https://doi.org/10.1021/ma00194a019
2. Cárdenas, G.; Cabrera, G.; Taboada, E.; Miranda, S.P. Chitin Characterization by SEM, FTIR, XRD, and 13C Cross Polarization/Mass Angle Spinning NMR. *J. Appl. Polym. Sci.* **2004**, *93*, 1876–1885. https://doi.org/10.1002/app.20647
3. Brunner, E.; Ehrlich, H.; Schupp, P.; Hedrich, R.; Hunoldt, S.; Kammer, M.; et al. Chitin-Based Scaffolds Are an Integral Part of the Skeleton of the Marine Demosponge Ianthella basta. *J. Struct. Biol.* **2009**, *168*, 539–547. https://doi.org/10.1016/j.jsb.2009.06.018
4. Pearson, F.G.; Marchessault, R.H.; Liang, C.Y. Infrared Spectra of Crystalline Polysaccharides. V. Chitin. *J. Polym. Sci.* **1960**, *43*, 101–116. https://doi.org/10.1002/pol.1960.1204314109
5. Focher, B.; Naggi, A.; Torri, G.; Cosani, A.; Terbojevich, M. Chitosans from *Euphausia superba*. 2: Characterization of Solid State Structure. *Carbohydr. Polym.* **1992**, *18*, 43–49. https://doi.org/10.1016/0144-8617(92)90186-T
6. Kolodziejski, W.; Klinowski, J. Kinetics of Cross-Polarization in Solid-State NMR: A Guide for Chemists. *Chem. Rev.* **2002**, *102*, 613–628. https://doi.org/10.1021/cr000060n
